# The perfect storm: A quadrifecta of complications with takotsubo cardiomyopathy

**DOI:** 10.21542/gcsp.2016.38

**Published:** 2016-12-30

**Authors:** Raj Amin, Timothy Fritz, Richard McNamara

**Affiliations:** 1 Michigan State University School of Human Medicine, USA; 2 Spectrum Health Frederik Meijer Heart & Vascular Institute, USA

## Abstract

Takotsubo cardiomyopathy is generally considered to have a favorable prognosis. However, several major problems may complicate the clinical course. We present a patient with four simultaneous complications of shock, left ventricular thrombus, left ventricular outflow obstruction, and severe mitral regurgitation. Despite this dramatic presentation, the patient had a complete recovery with resolution of all four complications within weeks.

## Introduction

Takotsubo cardiomyopathy (TTC), also called stress cardiomyopathy or apical ballooning syndrome (ABS), is an uncommon and fascinating form of acute transient left ventricular dysfunction in the absence of coronary artery disease. TTC commonly presents as an acute coronary syndrome, almost exclusively in postmenopausal women, and is frequently associated with acute emotional distress or chronic underlying anxiety and depressive states. The most common form is an akinesia of the apex combined with a hyperdynamic base resulting in a peculiar left ventricular (LV) configuration resembling a “takotsubo”, a Japanese octopus pot. The majority of patients have a favorable prognosis with full recovery of the left ventricular dysfunction. However, recent reports have suggested the prognosis is worse than conventional wisdom, with major cardiac and cerebral vascular morbidity and mortality, nearly that of acute coronary syndromes^1^. United States Medicare Registry shows a marked increase in the frequency of hospitalizations from TTS with a three-fold increase from the years 2000 to 2012^2^. The mechanism responsible for TTC and apparent increasing frequency are not completely understood.

Although in-hospital mortality is rare, several complications have been described in patients with TTC. These include arrhythmias, systolic anterior motion of the mitral valve (SAM), left ventricular outflow tract (LVOT) obstruction, mitral regurgitation (MR), LV thrombus, and cardiogenic shock^3–5^. Thus far, these complications have only been described in an isolated fashion. We present a unique patient manifesting four simultaneous complications of cardiogenic shock, severe LVOT obstruction with SAM, severe MR, and apical thrombus.

### Patient and methodology

A 59-year-old woman with a history of schizophrenia and anxiety presented with shortness of breath one week after treatment for community-acquired pneumonia. On exam, she was afebrile, hypotensive, tachycardic and had a 3/6 systolic murmur.

**Figure 1.**
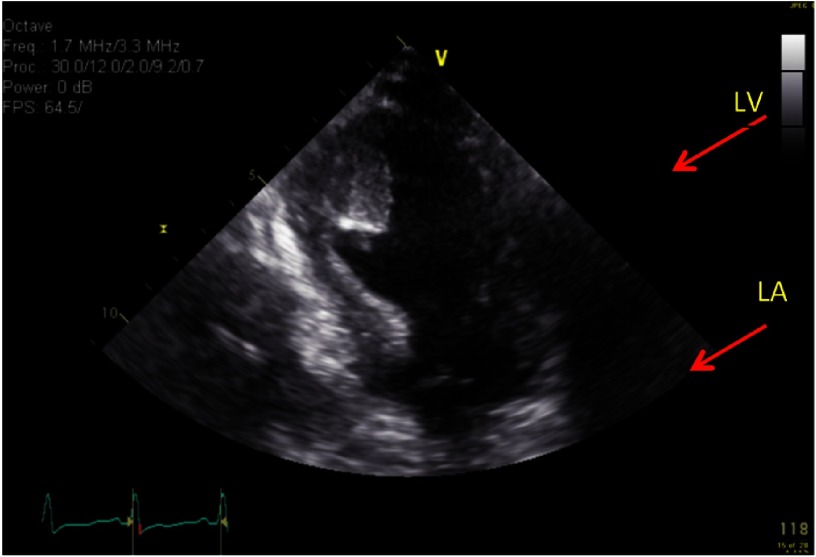
Apical two-chamber view showing thickened ventricular septum and apical clot.

**Figure 2.**
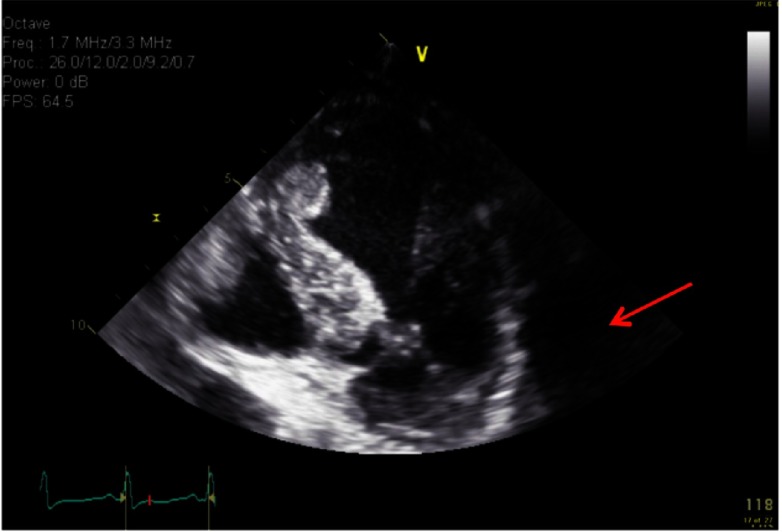
Echocardiography showing SAM of mitral valve with LVOT obstruction.

**Figure 3.**
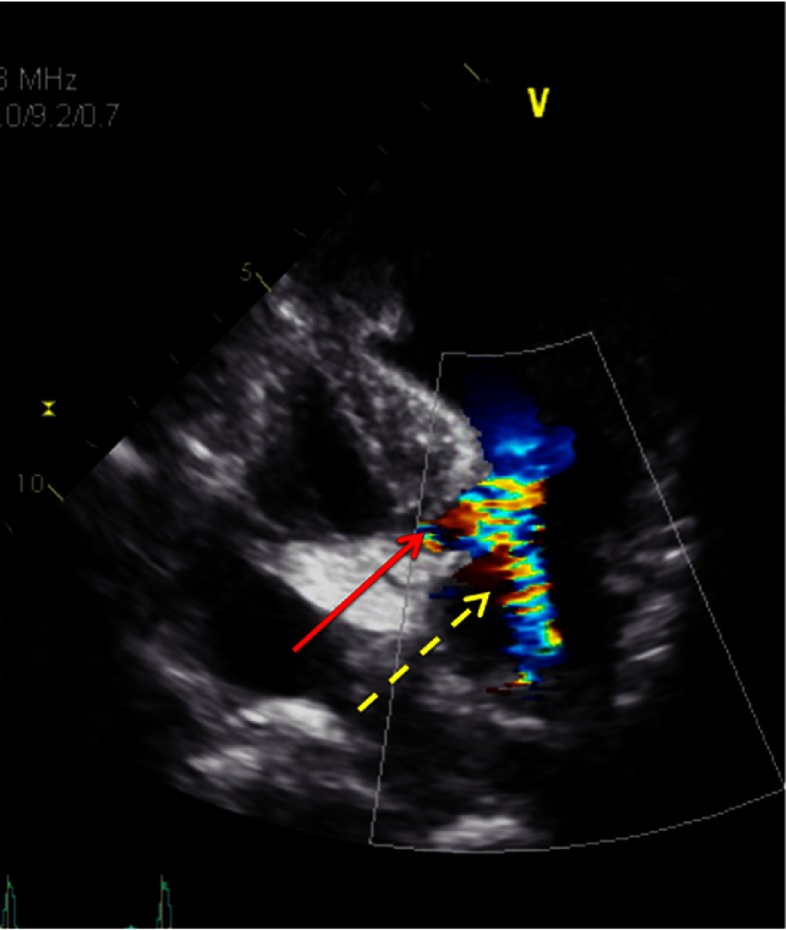
Echocardiography with Doppler showing severe mitral regurgitation and LVOT obstruction.

Laboratory studies demonstrated elevated Troponin I levels and an EKG revealed sinus tachycardia and diffuse T-wave inversions in the inferior wall and precordial leads. Echocardiography within 24 hours of admission noted classic signs of LV apical ballooning with hyperdynamic basal contraction, akinesis of the mid- and distal LV, and severely depressed LV ejection fraction (LVEF) of 15%. In addition, there was evidence of SAM of the mitral valve with LVOT obstruction (Figs. 2 and 3), and a large, apical LV thrombus measuring 2 by 4 cm (Fig. 1).

Repeat echocardiography within five days showed a marked improvement in LV function and a decrease in apical thrombus size, mitral regurgitation and LVOT gradient. Treatment included judicious fluid resuscitation, a beta-blocker and a heparin bridge to therapeutic Coumadin. A final echocardiography performed three weeks after admission showed a normal LVEF of 65%, trace evidence of mitral regurgitation, a fully resolved thrombus and normal movement of the left ventricular wall.

## Lessons learned

We present a patient with TTC and a perfect storm; four major complications at a single event of cardiogenic shock, severe LVOT obstruction with SAM, MR and apical clot. Despite an extremely complicated clinical presentation, we emphasize with patience and appropriate supportive care, full resolution of all major complications is possible.

## Competing interests

The authors of this article declined to declare any competing interests.

## LIST OF ABBREVIATIONS

LV: Left Ventricle
ABS: Apical Ballooning Syndrome
SAM: Systolic Anterior Motion
LVOT: Left Ventricular Outflow Tract
MR: Mitral Regurgitation
LVEF: Left Ventricular Ejection Fraction
TTC: Takotsubo Cardiomyopathy

## References

[1] Templin C (2015). Clinical features and outcomes of takotsubo (stress) cardiomyopathy. The New England Journal of Medicine.

[2] Murugiah K, Wang Y, Desai N, Spatz E, Nuti S, Dreyer R, Krumholz H (2016). Trends in short- and long-term outcomes for takotsubo cardiomyopathy among medicare fee-for-service beneficiaries, 2007 to 2012. Journal of the American College of Cardiology.

[3] Prasad A (2007). Apical ballooning syndrome: An important differential diagnosis of acute myocardial infarction. Circulation.

[4] De Gregorio C, Grimaldi P, Lentini C (2008). Left ventricular thrombus formation and cardioembolic complications in patients with takotsubo-like syndrome: A systematic review. International Journal of Cardiology.

[5] DeBacker O (2011). Tako-tsubo cardiomyopathy with left ventricular outflow tract (LVOT) obstruction: Case report and review of the literature. International Journal of Clinical and Laboratory Medicine.

